# Suicidal Ideation in Older Adults Who Highly Value Autonomy: The Role of Compensatory Strategies

**DOI:** 10.1093/geroni/igaf122.1213

**Published:** 2025-12-31

**Authors:** Catherine Ju, Amy Fiske

**Affiliations:** West Virginia University, Morgantown, West Virginia, United States; West Virginia University, Morgantown, West Virginia, United States

## Abstract

Greater value placed on one’s autonomy is related to suicidal ideation in older adults, but no research has examined the mechanisms underlying this relationship. The Motivational Theory of Lifespan Development contends that as individuals age and functional impairment increases, relying on one’s own effort may not be enough to obtain important goals. Therefore, older adults might use compensatory strategies (e.g., seeking help) to achieve their goals. However, older adults who highly value their autonomy may engage in fewer compensatory strategies, resulting in failure to achieve goals. This could contribute to feelings of hopelessness and suicidal ideation in older adults. This presentation will introduce research that examined whether (a) compensatory primary control strategies and (b) depressive symptoms mediated the relationship between value placed on autonomy and suicidal ideation and whether these effects were moderated by functional status in older adults. Older adults (N = 419) completed an online survey with questions about functional ability, value placed on autonomy, control strategy usage, depressive symptoms, and suicidal ideation. Results showed that compensatory primary control strategy usage and level of depressive symptoms partially mediated the relationship between value placed on autonomy and suicidal ideation. Unexpectedly, functional impairment did not moderate these relationships. Findings suggest that non-engagement in compensatory strategies is one reason value placed on autonomy is related to suicidal ideation in older adults. Investigating what may promote usage of these compensatory strategies could increase the field’s understanding of what factors lead to greater risk for suicide in older adults.

